# Exploring drivers and challenges in implementation of health promotion in community mental health services: a qualitative multi-site case study using Normalization Process Theory

**DOI:** 10.1186/s12913-018-2850-2

**Published:** 2018-01-24

**Authors:** Viola Burau, Kathrine Carstensen, Mia Fredens, Marius Brostrøm Kousgaard

**Affiliations:** 1 DEFACTUM – Public Health and Health Services Research, Central Denmark Region, Aarhus, Denmark; 2 Department of Public Health, University of Aarhus, Aarhus, Denmark; 3 The Research Unit for General Practice and Section of General Practice, Department of Public Health, University of Copenhagen, Copenhagen, Denmark

**Keywords:** Implementation, Community mental health services, Health promotion, Context, Normalization Process Theory

## Abstract

**Background:**

There is an increased interest in improving the physical health of people with mental illness. Little is known about implementing health promotion interventions in adult mental health organisations where many users also have physical health problems. The literature suggests that contextual factors are important for implementation in community settings. This study focused on the change process and analysed the implementation of a structural health promotion intervention in community mental health organisations in different contexts in Denmark.

**Methods:**

The study was based on a qualitative multiple-case design and included two municipal and two regional provider organisations. Data were various written sources and 13 semi-structured interviews with 22 key managers and frontline staff. The analysis was organised around the four main constructs of Normalization Process Theory: Coherence, Cognitive Participation, Collective Action, and Reflexive Monitoring.

**Results:**

Coherence: Most respondents found the intervention to be meaningful in that the intervention fitted well into existing goals, practices and treatment approaches. Cognitive Participation: Management engagement varied across providers and low engagement impeded implementation. Engaging all staff was a general problem although some of the initial resistance was apparently overcome. Collective Action: Daily enactment depended on staff being attentive and flexible enough to manage the complex needs and varying capacities of users. Reflexive Monitoring: During implementation, staff evaluations of the progress and impact of the intervention were mostly informal and ad hoc and staff used these to make on-going adjustments to activities. Overall, characteristics of context common to all providers (work force and user groups) seemed to be more important for implementation than differences in the external political-administrative context.

**Conclusions:**

In terms of research, future studies should adopt a more bottom-up, grounded description of context and pay closer attention to the interplay between different dimensions of implementation. In terms of practice, future interventions need to better facilitate the translation of the initial sense of general meaning into daily practice by active local management support that occurs throughout the implementation process and that systematically connects the intervention to existing practices.

## Background

People with severe mental disorders generally suffer from poorer physical health than the rest of the population in terms of comorbidity and mortality [1–5] and this also makes for poor quality of life [6, 7]. Various factors are considered to contribute to these increased risks including the side-effects of medication, access to health care, and lifestyle behaviours such as smoking, eating habits, and low levels of physical activity [1–5]. This has spurred an increased interest in developing and implementing health promotion interventions to improve the physical health of people with mental illness [1, 2, 4, 5, 8–10], many of whom are cared for in community mental health settings. There is rich literature on implementing various types of health interventions in community settings at large (for example [11–16]), but we know little about the specific implementation of health promotion interventions in adult mental health community organisations (but see [8, 9, 17]). Furthermore, the few existing studies have little concern for the effect of contextual factors on implementation, which is odd since contextual factors have been reported to be very important for implementing health interventions in community settings [11, 13, 18, 19]. Such interventions are typically complex and relatively loosely bounded and implementation emerges as highly contingent and context dependent [20–26].

Context can generally be defined as all factors that affect processes of organisational change but are not part of the intervention itself [21, 23]. Contexts for implementation in community settings are multi-level and nested [19, 27, 28]. Studies often distinguish between the external and internal contexts of organisations [24]. The former are understood as political, administrative and economic contexts in which organisations are embedded ([18, 28], more generally [20, 21]); the latter relate to specific formal and informal characteristics of individual organisations such as structure and culture ([19, 28], more generally [21]).

Against this background, the aim of the present study was to focus on the change process and to analyse the implementation of a structural health promotion intervention in community mental health organisations in different contexts in Denmark (for further details see the methods section below).

The study aimed to answer the following research questions:

1. How was the structural health promotion intervention implemented across different providers of community mental health services?
2. How did the organisational contexts of providers influence the implementation process?

The study used Normalization Process Theory (NPT) to analyse the implementation of the health promotion intervention in these different contexts [29, 30]. NPT has become a widely used theory for analysing the implementation of complex interventions and has previously been applied to a wide range of health topics and empirical settings including chronic health care, maternity care, e-learning and telemedicine [31]. A few NPT studies have investigated implementation in community mental health settings [32–35] and the implementation of health promotion interventions (see Bamford et al. on health promotion in nursing homes [36] and Sturgiss et al. [37] on a weight management programme in general practice). However, to our knowledge, no NPT studies have focused on health promotion interventions in community mental settings. Following NPT [30, 31], the process of implementing a complex intervention can be described and explained by employing four central theoretical constructs:

- *Coherence* refers to how and to what extend the relevant actors understand and make sense of the intervention; this includes whether actors can differentiate the intervention from previous practices; whether actors ascribe a positive value to the intervention; and whether actors understand what is expected from them.
- C*ognitive Participation* refers to how and to what extend actors engage themselves and others in driving the intervention forward; this includes issues of initiation, enrolment of relevant actors, and sustaining engagement.
- *Collective Action* refers to how - and to what extend - actors are able to enact the intervention in practice and how this enactment is connected to the allocation of skills and resources in the organisation and to existing practices and rules.
- R*eflexive Monitoring* refers to the way that the actors assess the consequences of the intervention individually and/or collectively and how these assessments may lead to new understandings and reconfigurations of the intervention.

Although the work of creating Coherence and Cognitive Participation are predominant during the planning of – or the earliest phases of – implementation [31], the relationship between the main theoretical constructs is not a linear one [29]. As implied by the concept of Reflexive Monitoring, NPT proposes that organisational actors are reflective so that their experiences during implementation affect their sense making (Coherence), their level of engagement (Cognitive Participation), and their ways of enacting the intervention (Collective Action). Thereby, NPT depicts normalization processes as dynamic and emergent [29].

## Methods

We used a qualitative multiple-case design and defined a case as a provider organisation of community mental health services that had implemented a specific structural health promotion intervention called *Sundere Liv i Socialpsykiatrien* (*Healthier living in community mental health services*) (SLIPS) [38]. In line with the literature on structural prevention in health care [39], SLIPS aimed at improving the health of users through activities that were embedded in and facilitated by changes in the structure of provider organisations including strategies to service delivery at multiple levels and the physical environment. This was based on the expectation that creating structural contexts that facilitate health promotion supports the healthy life style choices of individual users in a more sustainable way. SLIPS was designed as a complex intervention and the introduction of health promotion work packages relating to physical exercise, healthy eating and smoking reduction constituted the core of the intervention. The provider organisations included in the intervention had considerable leeway in defining specific activities under each health promotion package (see Table 1 below).

**Table 1:** Overview of the specific activities under each health promotion work package in selected providers of community mental health services, 2012

		Municipal provider Lakeside	Municipal provider Countryside^a^	Regional provider Beachfront	Regional provider Hilltop
Health promotion work package	Physical exercise	• Individual training programmes; • Groups for running/walking • Exercise buddies	• Daily/weekly walking tours	• Daily exercise • Groups for running/walking • Joint exercise day	• Spinning facilities • Outdoors exercise facilities
	Healthy eating	• Healthy meals/recipes	• Communal breakfast • Support shopping/meal preparation • Courses on healthy food	• Support shopping/meal preparation	• Courses on healthy food
	Smoking reduction	• Smoking cessation courses		• Dedicated smoking areas	• Dedicated smoking areas

^a^The provider did not have a health promotion work package on smoking reduction and suggested this was because of general resource constraints. However, this does not mean that the provider did not work with smoking reduction more generally; this is because the implementation of SLIPS coincided with the introduction of new anti-smoking legislation in public services

The implementation occurred between 2012 and 2015. The project secretariat was responsible for offering initial training to both managers and selected staff across the provider organisations involved in the intervention. This was completed by written guidance and on-going support based on monthly reports by the local provider organisations. Here local project groups were in charge of facilitating the process of defining local health promotion work packages and supporting their implementation. The authors were not involved in the implementation of SLIPS, but first conducted their study after the period of formal implementation had ended.

### Conceptual framework of the analysis

Our conceptual framework consisted of contexts (external and internal) of provider organisations and implementation activities. We first constructed an overview of context for our case selection. We focused on formal context factors and defined external contexts as the political-administrative structures of community mental health services and economic resources. We also included interorganisational relations [19, 24] as these had been an explicit element in the design of the intervention. We defined internal contexts as the formal characteristics of provider organisation, including formal structure and size, previous experience with health promotion, staff and other economic resources.

Implementation activities were operationalised in two stages. We initially defined implementation generally as different activity phases (preparation, implementation and sustainment) [24]. This provided the basis for our interview guide and a first, open coding of our interview data. We subsequently used the four basic theoretical constructs of NPT to code and analyse our data [40].

### Selection of cases

The SLIPS intervention included 24 provider organisations in total. The choice of provider organisations aimed at maximising variance to systematically assess how different external contexts affect implementation of health promotion in community mental health settings. The specific settings are complex and highly varied, especially in terms of political-administrative contexts. Community mental health services are offered by both regions and municipalities. Regional provider organisations are well established whereas municipal providers have entered the field more recently. Compared to their counterparts in the municipalities, regional providers function as a last resort and treat users with particularly complex needs. Conversely, municipalities enjoy considerable leeway in organising and funding community mental health services. Based on the differences in contexts our expectation was that regional providers compared to their counterparts in municipalities had more organisational and possibly financial resources for implementation.

The study included two regional providers (Beachfront and Hilltop) and two municipal providers (Lakeside and Countryside), which offered residential community mental health services. The differences in *political administrative structures* outlined above had repercussions for *interorganisational relations*: the integrated management of regional providers gave better possibilities to support interorganisational relations. In terms of *economic resources,* municipal budgets appeared to be under greater pressure during implementation; and we included two municipalities with different levels of economic resources, without being extremes. For an overview of the individual provider organisations, see Table 2 below.

**Table 2:** Overview of organisational characteristics of selected providers of community mental health services, 2012

	Municipal provider Lakeside	Municipal provider Countryside	Regional provider Beachfront	Regional provider Hilltop
Management structure	Part of Social, Health and Employment Section	Part of Social and Employment Section	Part of Section for Adult Community Mental Health Services	Part of Section for Adult Community Mental Health Services
	Municipal manager head of Disability and Mental Health Services	Municipal manager head of Centre for Psychiatry	Regional manager head of Section for Young People with Complex Needs	Regional manager head of Section for Substance Abuse and Huntington’s Disease
	One manager heads provider organisation	One manager heads several provider organisations	Two managers head provider organisation	Two managers head provider organisation
Users	Adults with severe, complex health problems and needs Often have both mental health and physical health problems Wide range of specific diagnoses and problems within and across provider organisations			
Size	32 staff	9 staff	40 staff	75 staff
	22 users	10 users	30 users	41 users

### Data collection

Data on external and internal contexts came from various sources, including literature on community mental health services, research reports, policy documents and statistics, documents specifically produced as part of the intervention, and supplementary interviews with the municipal/regional managers of community mental health services.

Data on implementation activities were generated through 13 semi-structured interviews with 22 key staff involved in implementation. Individual interviews were conducted with the managers of community mental health services in the municipalities/regions (4 interviews) and the managers of the provider organisations (4 interviews). Frontline staff was interviewed in groups (4 interviews); this was except in one case where a staff member was unable to participate in the group interview (1 interview). The choice of respondents was partly given, as there was only one manager of community mental health services and the provider organisation respectively in each case. Involvement of frontline staff was restricted by a combination of availability and provider resources, as the time for interviews was part of normal work shifts. Where possible, respondents with direct experience with implementation (for example from a project group) were given prioritity. The researchers were assisted by either the managers of the provider organisations or the municipal/regional managers in identifying relevant respondents among the staff. This also helped to ensure there was sufficient recruitment of frontline staff. All managers agreed to be interviewed.

The interviews lasted 30–40 min and were conducted in early summer 2015. The first three authors conducted the first four interviews together to ensure consistency of the subsequent interviews, which they conducted individually. Based on our initial operationalisation of implementation activities according to different phases (for more details see section on ‘Conceptual framework of the analysis‘), we developed an interview guide to cover the following themes: 1) planning for new health promotion practice (*preparation*), including formulating health promotion packages, who was involved and how, and what challenges were encountered; 2) introducing new health promotion activities (*implementation*), including how implementation was approached, how the new practices were experienced, and what challenges were encountered; 3) maintaining services (*sustainment*), including plans for sustainment and delegation of responsibility.

### Data analysis

Following the two-stage operationalisation of implementation activities (see section on ‘Conceptual framework of the analysis’ above), the analysis began with an open coding process to identify key themes of the implementation process. This was subsequently used to construct a set of codes derived from the operationalisation of the NPT framework. In relation to both rounds of coding, all authors independently performed preliminary coding of selected interviews and following a discussion among the authors the themes and codes were refined and settled. We analysed the interview material using NVivo 10 software based on a thematic approach that combined deductive and inductive elements to identify common threads [41]. The coded material was discussed among all authors and then collated to create preliminary themes, which were subsequently reviewed and refined. We first conducted a within-case analysis, followed by a cross-case analysis. We did this both individually and collectively, and the iterative work method resulted in a joint analysis.

## Results

The analysis examines the implementation process across the four providers based on four NPT-dimensions of implementation. Following the expected importance of context, the analysis pays particular attention to distinct patterns of implementation across individual providers and between municipal and regional providers.

### Coherence

Across providers, staff perceived SLIPS to be based on a broad understanding of health. The focus was on health behaviour and more general lifestyle patterns of users. This included acknowledging the complex and heterogeneous circumstances and needs of users, such as different age/weight issues, varying severity of mental illness and different ambitions regarding own health. During implementation, this translated into a question about what gave physical and mental wellbeing and this included factors related to lifestyle diseases and tools to develop personal skills to manage life, wellbeing, and health in a better way:

‘[O]ne could say that this [SLIPS] of course concerns lifestyle factors of diet, smoking, alcohol consumption and physical activity, but this [SLIPS] is also about developing personal skills to cope with one’s life. […] [A]bout trying to work with educational issues around medicine use, with getting a better grip on one’s life and other related issues. This corresponds with our understanding of health, which is rather broad [...]’ (Manager, Municipal Provider Lakeside)

Across providers this understanding of health also connected well to ‘recovery’ as the predominant treatment approach in community mental health services:

‘We saw this [SLIPS] as part of work we are already doing and we want to continue to focus on […]. […] [P]art of our frame of reference is also that we work based on ‘recovery’. We should facilitate rehabilitation, so that the individual user [...] can experience some [personal] development and recover [from illness] to some extent. From this perspective, we felt that this [participating in SLIPS] was only natural.’ (Regional Manager, based in Regional Provider Beachfront)

Health and health promotion emerged as integral components of the ‘recovery’ approach. The corresponding practice was concerned with supporting personal development towards a ‘better’ life as defined by individual users and through a wide range of activities such as weight loss, smoking cessation and social activities.

Most staff respondents across providers considered SLIPS to be in line with earlier and parallel health promotion initiatives. Continuity generated recognisability and offered concrete experiences to draw on during implementation of SLIPS. For example, Municipal Provider Lakeside had learned from earlier initiatives that involving staff with special enthusiasm for a project greatly enhanced implementation; under SLIPS, the provider focused on working with ‘change agents’. The staff at Regional Provider Hilltop felt that SLIPS had positive knock-on effects on health promotion activities because it helped keep existing activities on track and allowed them to develop activities in this area. The staff also suggested that continuity reinforced their perception of SLIPS as meaningful and that this had a positive effect on their motivation to engage in implementation. However, this perception was not shared by all staff. Some staff at Municipal Provider Lakeside found SLIPS to be very similar to previous projects in their organisation and felt that they were already well ahead of other participating providers when SLIPS was introduced. Therefore, SLIPS became less meaningful to them which affected their motivation in a negative way. Thus, the consequences of continuity in relation to earlier/parallel health promotion initiatives appeared ambivalent suggesting a tipping point where ‘too much resemblance’ (as perceived by participants) can impede implementation. This is captured by the NPT sub-construct of Differentiation which emphasises that participants should be able to distinguish a newly adopted intervention from previous ways of working [42, 43].

### Cognitive participation

The cognitive participation of managers initially included a) decisions to adopt SLIPS and formulating health work packages; b) choosing individual health work packages and defining specific activities; and c) defining the project organisation. Managers at the individual providers generally took the lead on the first set of activities (except from Municipal Provider Countryside where the municipal management was the prime mover and the provider management only offered feedback). The remaining two activities were delegated to project groups, which also included frontline staff.

Management involvement in subsequent implementation work varied. At Municipal Provider Lakeside and Regional Provider Beachfront frontline staff experienced that both municipal/regional management and the provider management were involved throughout the entire implementation process, for example by keeping a continuous focus on SLIPS at staff meetings. At Municipal Provider Countryside and Regional Provider Hilltop managers appeared to be less engaged in terms of securing internal support through enrolment and legitimation. Frontline staff perceived this sporadic involvement as a lack of support.

As for staff, problems with cognitive participation were – to a varying extent – present among all providers. The respondents offered several explanations for resistance or lack of engagement: some staff felt that health promotion activities overstretched users’ resources and thus had a negative impact on their quality of life; others argued that health promotion activities did not respect personal preferences of users and staff; and some felt that too many things were happening at the same time.

One of the important implementation ideas in the SLIPS was the concept of staff being role models for health promotion. As role models staff was expected to participate in different health promotion activities (like joining users for walks and meals) and to display a healthy lifestyle at work. In the four providers, such expectations were formulated and formalised by management or by key implementation staff to different extents. However, in all cases some staff did not buy into this idea; they felt that the elements of smoking cessation and healthier meals interfered with their usual lifestyle and personal preferences:

Frontline staff 1: ‘[W]e had fried fish with shrimps on white bread [a traditional Danish dish], so there has to be some dressing on top, sorry!’

(Frontline staff, Municipal Provider Lakeside)

Over time, however, most staff came to accept being role models stressing that it was about aiming for consistency:

‘[T]his is about being role models; if we [as staff] cannot change, then we cannot expect our users to change.’ (Frontline staff, Municipal Provider Lakeside)

### Collective action

Across providers, staff based health promotion activities on an approach of ‘gentle motivation’ and aimed at small incremental changes by motivating users through dialogue and involvement rather than imposition:

‘[T]alking with users. That is the most important thing [...]. [To find out] what motivates them? Otherwise we can jump to the end of the world and still not succeed. [...] [T]he most important thing is what the user wants. […] [T]o start with small [day-to-day] changes and to recognise this as a success. And to start the [personal] development from there […].’ (Manager, Regional Provider Hilltop)

Regional Provider Hilltop also used nudging techniques such as placing the healthiest food on green plates at the front of the buffet. Municipal Provider Lakeside initially adopted a more ambitious approach, formulating more restrictive menu rules and banning staff traditions of celebrating important personal/social events with cake. However, this backfired and led to considerable resistance among frontline staff and users.

Across providers, staff presented the incremental approach as a response to the complex circumstances, behaviours and needs of a heterogeneous group of users which posed several challenges to enacting the intervention. Some users displayed externalised aggressive behaviour; some were underweight, others severely overweight; and some were afraid to leave their apartment. It was often difficult for staff to involve a sufficient number of users in group activities, which had to be supplemented with individually tailored activities. This required motivational skills, a high level of engagement, considerable flexibility and a lot of time:

‘This makes considerable demands on staff as they always have to be flexible and identify the [specific] situation users are in and what their needs are; and what kind of activity is most suitable [under the given circumstances].’ (Regional manager, based at Regional Provider Beachfront)

Staff also needed to show enthusiasm for health promotion to the users by participating regularly in specific intervention activities:

‘Interviewer: [...] [I]nvolvement of staff, that they show up [for health promotion activities] is critical for the users showing up?

Regional manager: Yes. And if you cancel the walk twice, you can start from scratch [...]. Then they [users] do not believe that [the walk] will go ahead and they [users] become less engaged. Therefore, it is so important that activities [actually] take place.’ (Regional manager, based at Regional Provider Hilltop)

Planning was key to ensuring that staff was always available for activities, but it was also important to be flexible to meet individual needs. Here, time was mentioned as a barrier to implementation. For example, some frontline staff at Municipal Provider Lakeside did not always feel that they had time to motivate users, and frontline staff at Regional Provider Beachfront sometimes had difficulties finding time to take part in healthy activities such as going for a walk instead of driving. Staff at the other two provider organisations also mentioned insufficient financial resources as a barrier to planning and delivering the intended health promotion activities.

Overall, and relating to several NPT-constructs, the implementation process was highly sensitive to changes in the user group as well as among frontline staff, especially in cases of staff turnover due to long-term sickness, maternity leave, or resignations:

‘[C]oncerning the [health promotion] activities, we have to be highly alert that [activities] do not peter out, when one of our staff stops or also more generally when our users [leave]. [We have to ensure] [t]hat there is new blood who can take over.’ (Frontline staff, Regional Provider Hilltop)

When key staff left, the providers lost valuable knowledge and dedication, and they constantly had to explain the underlying rationale of SLIPS to new frontline staff (Coherence), encourage new staff to involve themselves in health promotion activities (Cognitive Participation); and in some cases, to upgrade the qualifications of new staff so that they would fit in to the existing allocation of work (Collective Action: Skill-set workability).

### Reflexive monitoring

Formal monitoring systems were in place as soon as implementation began. Project teams had to complete a report to management every other month. The aim was to document local implementation processes, based on, among other things, the Plan-Do-Study-Act Worksheet, and to make it possible to share experiences across providers. The material available on SLIPS’ internal home page suggested that providers only used the formal monitoring systems to a limited extent. Providers completed between none to four reports in contrast to the expected five to seven reports. In many of the reports, multiple sections were left blank. Finding time to meet was highlighted as a critical barrier to working together to evaluate progress and results (Communal appraisal):

‘The biggest barrier remains that we have been unable to meet as a [project] team and do the necessary follow-ups.’ (Municipal Provider Countryside, 2-monthy report, August 2013)

In general, Reflexive Monitoring tended to become informal and ad hoc because it had to fit into the staff’s busy work days:

‘[B]ecause of Christmas it has been difficult to find time to meet. However, there has been on-going dialogue among [members of] the team via email and when we have bumped into each other in the hallway’ (Regional Provider Hilltop, 2-monthly report, March 2013)

Regional Provider Beachfront seemed to have the most formalised approach to monitoring, which was firmly embedded in meetings among staff and with users:

‘[T]he project [SLIPS] is discussed every Monday at morning coffee [with staff] […] [and] changes in practice are evaluated as part of meetings with residents […].’ (Regional Provider Beachfront, 2-monthly report, January 2013)

However, despite these formal elements, monitoring was also mostly informal at Regional Provider Beachfront. This informal and ad hoc approach to monitoring, which characterised all providers, also applied to adjusting the activities. In their daily work, frontline staff continuously evaluated and adjusted activities based on informal input by users and staff. An important criterion was changes in users’ interest in or resources for participating in certain activities:

‘[Y]ou could say that the initiatives we have taken concerning exercise are changing all the time. Because this is about trying out something based on the people who live here at present. Does this [specific activity] make sense? And this changes a lot.’ (Regional manager, based at Regional Provider Beachfront)

Adjustments were also made when staff involvement in facilitating certain activities changed. Staff across providers acknowledged that the informal and ad hoc approach to monitoring and adjustment of activities to a large extent reflected challenges posed by the users and their characteristics (see section on Collective Action above).

## Discussion

This study investigated how a structural health promotion intervention was implemented by different providers of community mental health services and how the organisational contexts of regional and municipal providers influenced implementation. We analysed the implementation process using the four main theoretical components of NPT; Table 3 below gives an overview of our findings.

**Table 3:** Overview of findings

Coherence • Existing understandings of health and health promotion • Perceived connections to existing goals and practices	SLIPS perceived as meaningful across providers • Based on broad understanding of health related to what gave physical and mental well-being • Integral component of ‘recovery’ as predominant treatment approach; Continuity with other health promotion initiatives had ambivalent impact
Cognitive Participation • Engagement in implementation process	• Engagement of management varied; From integrated and continuous involvement to decoupled and ad hoc • Engagement of staff was an important challenge; Some did not buy into the concept of being role models
Collective action • Enactment of the intervention: Approach to working with health promotion • Importance of user and staff resources	• Approach of ‘gentle motivation’ across providers • Enacting the intervention in interactions with users was challenging due to the heterogeneous user group with complex circumstances and needs; Required high level of staff engagement and flexibility
Reflexive monitoring • On-going monitoring and adjustment of health promotion activities	• Informal, ad hoc monitoring and adjustment of activities predominant across providers

There are few studies of implementing health promotion in community mental health services [8, 9, 17] and they mostly focus on general characteristics of the implementation such as different stages of the implementation process and the adaptation to the resources of provider organisations. With its more detailed analysis of the drivers and challenges in the implementation process itself, the present study thus makes a needed empirical contribution. As discussed below, the study also makes a more general contribution: firstly, NPT allows for a more integrated perspective on the connections between different dimensions of implementation work; secondly, the study underscores the limitations of using pre-defined context characteristics.

Our findings under *Coherence* pointed to positive conditions for implementation. Most respondents found the SLIPS intervention meaningful although this was mainly expressed in general terms. Staff characterised SLIPS as a ‘natural’ extension of existing work with health promotion in community settings and referred to a positive fit with the notion of ‘recovery’ as the predominant treatment approach in community mental health services. In three providers, this seemed to support initial adoption, echoing findings in the literature that a sense of continuity facilitates organisational change [19]. However, staff in Municipal Provider Lakeside felt that SLIPS was yet another health promotion intervention and that it did not introduce anything new and these problems with differentiation weakened their motivation.

While most respondents across providers generally found the ideas and principles of SLIPS to be meaningful, the *Cognitive Participation* of managers and staff was more mixed. Management was involved in a variety of ways, formulating health promotion work packages at the beginning and supporting frontline staff in specific implementation activities. However, there were distinct variations; for some managers, the engagement was integrated and continuous, for others it was decoupled and ad hoc. This is noteworthy since active leadership has been identified as crucial during implementation of new practices in community mental health settings. For example, in relation to integrated treatment for people with mental health and substance abuse problems, Bonham et al. [11] suggest that leadership is one of two central factors influencing the capacity of community-based provider organisations to implement the use of evidence based practice. Leadership also emerged as the most influential factor in Whitley et al.’s [44] study of implementing an illness management and recovery programme. Torrey et al. [45] further argue that active leadership is especially effective when focused on redesigning the work flow, that is: reworking policies, documentation and meeting structures as well as supporting staff functions.

There were similar variations in the *Cognitive Participation* of frontline staff. It was a central element in the implementation of the intervention that staff acted as role models for the users. Some staff was critical towards this concept as they felt that it interfered with their lifestyle and personal preferences. This scepticism was apparently gradually overcome by the argument that being role models made it more legitimate for frontline staff to engage with users about health promotion. Still, staff engagement in specific implementation activities was mixed due to either user concerns or provider specific circumstances in the implementation process. This is problematic, as active support by staff is important for engaging people with severe mental illness in lifestyle interventions as Roberts and Bailey’s [1] have shown in a literature synthesis.

As for *Collective Action*, an approach of ‘gentle motivation’ was predominant across providers as they operationalised the intervention. Still, enacting the intervention in daily interactions with users was difficult due to the varied needs and capacities in a complex and changeable user group. Staff had to provide ongoing motivational and practical support. This involved: considering the individual situation of users; planning and performing regular participation in activities; and continually adjusting activities based on user responses. Such challenges of engaging mentally ill persons in health promotion are congruent with the findings of McKibbin et al. [17]. The authors report that community mental health providers experienced that ‘psychiatric symptoms, poor cognitive functioning, physical health problems, and limited health knowledge’ ([17], p. 573) inhibited user abilities to make healthy life style changes.

Finally, practices of *Reflexive Monitoring* in all providers were characterised as being informal and ad hoc. While this served to make ongoing adjustments to activities, it may be viewed as insufficient for systematic implementation of this kind of multifaceted intervention. For example, Torrey et al. [45] found that measurement and feedback is a core ingredient of active leadership and as such strongly influences successful implementation.

Overall, both practical implementation efforts and ongoing evaluations of the intervention to a considerable extent depended on motivated and competent staff and were highly vulnerable to limited time resources and changes in staffing and staff turnovers, which is a common challenge in community services [11, 45]. For instance, in their study of evidence based practice for integrated treatment of co-occuring mental health problems and substance abuse, Bonham et al. [11] identified the availability of financial resources as the overriding influence on uptake.

As it appears from the above, some aspects of our findings are echoed by other studies [1, 11, 17, 19, 44, 45], but the present study is distinct as it used NPT as a framework for analysing implementation. Thereby, our study also makes a more general contribution to the literature on the implementation of health promotion in community mental health care and similar complex interventions in community mental health settings. Using NPT offered a more integrated perspective and highlighted the connections and disconnections between different dimensions of implementation work. For example, the relative high level of Coherence in most providers concerning the structural health promotion initiative offered favourable conditions of implementation, but this did not easily translate into Cognitive Participation and Collective Action. These disconnections pointed to the challenges for implementation associated with the organisational context of providers of community mental health services. This not only included the complex groups of users but also the staff at community mental health providers, who are different from hospital settings. A substantial share of staff has professional backgrounds in education, and staff with health background is typically only trained at helper/assistant level [46]. Hence, staff may lack skills in health promotion, and this can have repercussions for implementation. Somewhat similar Himelhoch et al. [8] found that staff with professional backgrounds in psychology falsely believed that users were not interested in quitting smoking and that this constituted a major barrier to smoking cessation in community mental health. Studies in Denmark have also shown that staff attaches important positive functions to smoking [47, 48], such as quality of life and leverage for therapeutic encounters. These factors may explain why for some frontline staff in SLIPS the relationship between seeing health promotion as meaningful and engaging in specific health promotion activities was complex and created some resistance.

The prominence of contexts that were common to all providers (as they related to the broader characteristics of the field of community mental health services) seems to be at odds with literature where the main concern is the importance of *different* contexts for implementation in community settings [11, 13, 18, 19]. Based on this, we expected systematic differences in patterns of implementation across providers due to differences in external contexts, and that regional providers would find implementing health promotion in community mental health services easier than their counterparts in the municipalities. However, the variations we found, did not systematically relate to particular contextual characteristics of municipal and regional providers. Variations were also secondary compared to the strong similarities in implementation challenges across providers. This is an interesting finding and requires further research as we suggest below.

### Methodological limitations

The study included only four provider organisations, and systematic differences between municipal and regional providers would possibly have emerged more clearly if we had included more providers. Alternatively, we could have paid more attention to the organisational characteristics of providers as another dimension of context by including non-residential services such as municipal day centres and supported housing (where users may have less complex needs).

The study applied qualitative interviews as the primary data collection method. This entails some limitations regarding the level of insight that can be gained about daily implementation processes compared to participant observations. Unfortunately, within our budget it would have been too costly for the study to perform participant observation in all four organizations for an extended period of time. We also did not collect any detailed demographic data about the persons interviewed and this limits the sense of the representativeness of our sample. Furthermore, it is a limitation that the study only included the perspectives of managers and staff since interviews with the users of the provider organisations might have produced a more complete picture of implementation dynamics.

NPT generally offered a useful way of organising our data and we did not find important themes that did not fit into the framework. However, we did experience difficulties with coding and reporting some of the data without overlap between constructs (cf. [31]), particularly in relation to the constructs of Cognitive Participation and Collective Action.

### Implications for research

Our analysis points to the importance of contexts related to the specific characteristics of users and staff, in that these contexts challenge the implementation of health promotion in community mental health services. There can be systematic variations in the composition of users and staff and future studies of context in community mental health services should adopt a more bottom-up, grounded descriptions of context to identify the specific aspects that affect implementation of health promotion interventions. Developing such a description could be part of preliminary interviews with management and staff. As far as applying NPT to study the implementation of health promotion in community settings, our study suggests that future research should pay closer attention to the complex interplay between the four dimensions of implementation work; for example, how the interplay between the intervention, the contextual integration efforts of management and staff characteristics mediate how coherence is translated into engagement.

### Implications for practice

Our study shows that the fact that staff considers health promotion meaningful does not necessarily translate into engaged, collective implementation efforts. Instead this requires the explicit attention of management and the following three points concerned with strengthening the role of management may facilitate implementation of health promotion interventions in community mental health services. First, to retain staff motivation, management must ensure that staff has a clear understanding of how the intervention should be integrated with existing practices. Second, management must engage in the implementation process not only initially but throughout the process to support staff engagement by showing that health promotion is prioritised in resource allocation decisions. Third, management needs to focus on supporting the hands-on participation in daily implementation work.

## Conclusion

Despite broad interest in health promotion to improve the physical health of people with mental illness, little is known about the implementation of such interventions in adult mental health community organizations and the role played by contextual factors. Here the present study contributes with new qualitative knowledge by identifying key drivers influencing the implementation of a structural health promotion intervention in community mental health organisations in different contexts. The study showed that while staff generally found health promotion meaningful, their engagement was mixed and included some resistance. Particularly, the complexity of the user group (in regard to problems and needs) emerged as an important challenge to implementation. Other characteristics of the organisational context common to all providers, such as the occupational background of staff, also seemed important for implementation. It was somewhat surprising that no differences in implementation dynamics could be directly ascribed to the influence of traditional context factors such as external political-administrative structure and organizational size. The implications are for researchers to develop a more bottom-up understanding of context and for intervention teams to better facilitate the process of meaningfully converting positively received principles of health promotion into continuing daily work processes. This requires active local management involvement throughout the implementation process in terms of enrolling staff and supporting staff with connecting new health promotion initiative to existing practices.

## Abbreviations

NPT: Normalization Process Theory
SLIPS: *Sundere Liv i Socialpsykiatrien* (*Healthier living in the community mental health services*); name of the health promotion intervention studied
